# Expression of AKT1 Related with Clinicopathological Parameters in Clear Cell Renal Cell Carcinoma

**DOI:** 10.3390/cimb44100334

**Published:** 2022-10-15

**Authors:** Taesoo Choi, Koo Han Yoo, Man S. Kim

**Affiliations:** 1 Department of Urology, School of Medicine, Kyung Hee University, Seoul 05278, Korea; 2 Department of Biomedical Science and Technology, School of Medicine, Kyung Hee University, Seoul 05278, Korea; 3 Translational-Transdisciplinary Research Center, Clinical Research Institute, Kyung Hee University Hospital at Gangdong, Seoul 05278, Korea

**Keywords:** carcinoma, renal cell, AKT1 protein, survival analysis

## Abstract

Pathways such as VEGF, EGF and mTOR are known to be one of the major mechanisms of tumorigenesis including kidney cancer. To identify potential signaling pathway proteins, we performed differential/correlation analyses of mTOR-associated genes from three public datasets. AKT1 protein, one of the PI3K/AKT/mTOR pathways, turned out to be the potential by showing a consistent discrepancy between ccRCC-associated conditions as well as strong correlation with other mTOR-associated genes across the datasets. Then, we analyzed how AKT1 alteration affects clear cell renal cell carcinoma. The pathology of 58 kidney cancer patients was constructed to analyze the relationship between the expression level of AKT1 through immunohistochemical staining and their clinicopathological data. Gender, age and TNM stage did not show significant results. AKT1 is a known oncogene. However, in this study, high expression of AKT1 showed a slight correlation with lower WHO/ISUP grade, longer recurrence-free and progression-free survival rates.

## 1. Introduction

Renal cell carcinoma (RCC) accounts for 2%–3% of malignant tumors in adults. About 64,000 new RCCs are diagnosed each year in the United States, and 14,400 patients die of RCC [1]. Most of RCC is sporadic, and only 4%–6% are thought to be familial [2]. The incidence of RCC has increased by an average of 3% per year since the 1970s, which is thought to be due to the wide use of abdominal CT or ultrasound [3].

RCC is generally known to occur mainly in proximal convoluted tubules, which is pathological mechanism of clear cell and papillary type renal cell carcinoma. It has a limited response to cytotoxic anticancer drugs, so patients with metastatic disease usually have a poor prognosis. Emerging medications to treat metastatic RCC have been developed through elucidation of VEGF, mTOR and related immune regulatory pathways [4].

AKT1, contained as a part of PI3K/AKT/mTOR pathway, is involved in cell survival pathways such as inhibiting apoptosis, angiogenesis, cell migration and invasion [5]. It is known that the over-activation of the PI3K/AKT/mTOR is associated with the development and progression of tumors through cell hyperproliferation, infiltration, and resistance to treatment [6,7]. Subtypes of AKT, such as AKT1, AKT2, and AKT3 are all supposed to have their own important roles [8].

The goal of our study was to compare and analyze the gene regulation of AKT1 protein, clinical characteristics and survival rate of clear cell RCC. There are few pieces of research on the relationship between AKT1 expression and clear cell RCC, to the best of our knowledge, our study is the initial report on this theme.

## 2. Materials and Methods

### 2.1. Evidence Acquisition via Analysis of Three RNA-Seq Datasets

Two clear cell RCC-based mouse model datasets, available under the accession GSE131735 and GSE199795, and a human dataset from clear cell RCC patients, published under the accession GSE175648 were downloaded from GEO (Gene Expression Omnibus). Expression patterns of mTOR-associated genes whose gene list were obtained from [9] were compared between all three datasets by z-score and visualized using Circlize. Furthermore, differential analysis of mTOR-associated genes was implemented through DESeq2 [10] and illustrated using EnhancedVolcano. Furthermore, correlation analysis was carried out through Pearson correlation and its visualization was completed using Cytoscape [11].

### 2.2. Clinical Tissue Samples

Tissue samples were acquired from 58 cases of clear cell RCC that were surgically resected at Kyung Hee University Hospital at Gangdong from January 2006 to December 2013. Tumors were staged according to the criteria of the American Joint Committee on Cancer [12]. The clinical parameters including tumor grade, recurrence, progression and overall survival, were evaluated retrospectively.

### 2.3. Immunohistochemical Staining and Analysis

Immunohistochemistry was performed on 4-µm tissue sections from each tissue block using the Bond Polymer Intense Detection system (Vision BioSystems, Victoria, Australia). In brief, 4-µm sections of formalin-fixed, paraffin-embedded tissue were deparaffinized with Bond Dewax Solution (Vision BioSystems, Victoria, Australia), and an antigen retrieval procedure was performed using Bond ER Solution (Vision BioSystems, Victoria, Australia) for 30 min at 100 °C. The sections were incubated for 15 min at ambient temperature with primary polyclonal antibodies to AKT1 (1:200, Abcam, Cambridge, UK) using a biotin-free polymeric horseradish peroxidase-linker antibody conjugate system in a Bond-max automatic slide stainer (Vision BioSystems, Victoria, Australia). Fine images were captured digitally using the DXM 1200 camera system (Nikon, Tokyo, Japan). Immunohistochemical staining for AKT1 was evaluated based on intensity and proportion. The intensity score was defined as 0 (no staining), 1 (weak staining), 2 (moderate staining), and 3 (strong staining). The proportion score was defined as 1 (<30% of tumor cells) and 2 (≥30% of tumor cells). The intensity score and proportion score were multiplied together for a total score. Total scores were as follows: 0–1 (low expression) and 2–6 (high expression).

### 2.4. Statistical Analysis

Using Version 20.0 IBM SPSS statistics program, Fisher’s direct probability method was used to confirm the relationship between the protein expression level of the AKT1 and the clinical characteristics of the patient. Kaplan–Meier test was used to form a recurrence curve of renal cancer patients and log-rank test was used to confirm the association according to the difference in protein expression level of the AKT1. When the *p* value was less than 0.05, it was defined as statistically significant. Furthermore, multivariate survival analysis with Cox proportional hazard model was applied to analyze how survival time of patients was associated with other variables such as age, sex, Fuhrman nuclear grade, T stage, N stage, M stage, recurrent, survival, and progress. The multivariate analysis was visualized by Forest plot.

## 3. Results

### 3.1. Evidence Synthesis

We brought into focus on the underlying interconnection mechanism between mTOR signaling pathway and RCC since it is conservatively known that mTOR pathway is recurrently involved in RCC through proliferation or survival of cancer cells. We analyzed and visualized the relative expression levels of mTOR-associated genes from three distinct ccRCC-associated public datasets, (i.e., RNA-seq datasets); (i) GSE131735: WT vs. mutation (Vhl and Tsc1 loss) from kidney-specific deletion of Vhl and Tsc1 in mice; (ii) GSE199795: WT vs. somatic mutation (Bap1, Pbrm1 and Setd2) in genetically engineered mouse models; (iii) GSE175648: progressive vs. non-progressive intermediate/high-risk clear cell RCC in patients.

**Figure 1:**
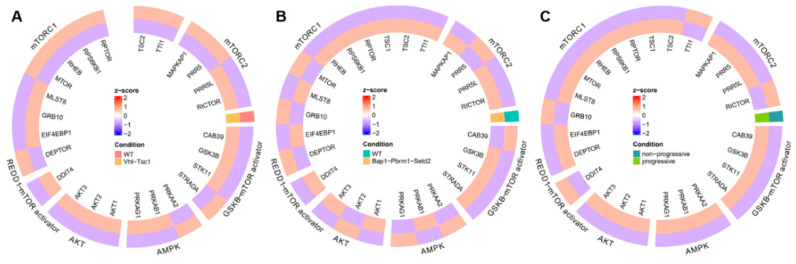
Expression levels on mTOR-associated genes across three distinct datasets. (**A**–**C**) The circular heatmaps display relative expression levels for three different datasets ((**A**): GSE131735, (**B**): GSE199795, (**C**): GSE175648), where z-scores of each gene are used for comparison.

We first explored relative expression levels of mTOR-associated genes across the three datasets through their z-scores as shown in Figure 1. mTOR pathway is composed of six modules and the list of mTOR-associated genes is as follows; (i) AMPK: PRKAA2, PRKAB1, and PRKAG1; (ii) GSK3B-mTOR activator: GSK3B, STK11, CAB39, and STRADA; (iii) REDD1-mTOR activator: DDIT4; (iv) AKT: AKT1, AKT2, and AKT3; (v) mTORC1: TSC1, TSC2, RHEB, RPTOR, AK1S1, MTOR, DEPTOR, MLST8, TEL2, TTI1, CLIP-170, GRB10, LIPIN1, ATG1, EIF4EBP1, and RPS6KB1; (vi) mTORC2: MAPKAP1, RICTOR, PRR5, and PRR5L. Eight genes such as AKT1, AKT3, EIF4EBP1, MLST8, PRR5L, GSK3B, STK11, and PRKAB1 indicated consistent upregulation patterns across all the datasets. Furthermore, neither mTOR-associated modules demonstrated the same up/down regulation pattern nor no genes were constantly down regulated over the datasets.

**Figure 2:**
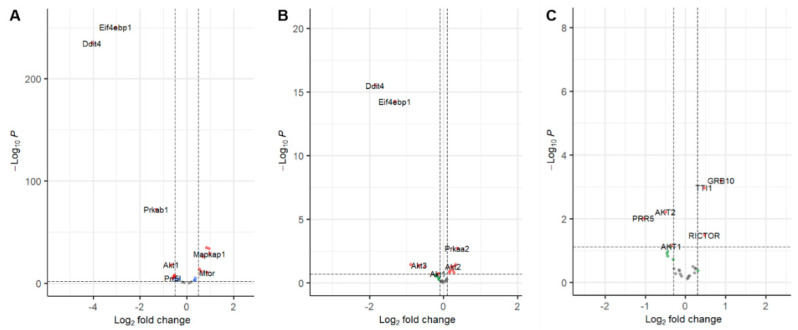
Differential analysis on mTOR-associated genes across three distinct datasets. (**A**–**C**) Volcano plots for significantly differentially expressed mTOR-associated genes from three different datasets ((**A**): GSE131735, (**B**): GSE199795, (**C**): GSE175648).

In order to assess the effects of mTOR-associated genes on the three different comparisons, we applied volcano plots to identify significantly differentially detected genes for each dataset as illustrated in Figure 2. On average, around 30% of mTOR-associated genes turned out to be significantly expressed between two different conditions of each dataset. DDIT4 and EIF4EBP1 displayed similar significant differential regulation for the two mouse models (A: GSE131735 and B: GSE199795), while AKT2 showed the abnormal discrimination between the mice (B: GSE199795) and the patients (C: GSE175648). Although having unimpressive significance, only AKT1 demonstrated differential expres-sion levels across all three datasets by having statistics as follows: (i) GSE131735: logFC (4th) and *p*-value (4th), (ii) GSE199795: logFC (6th) and *p*-value (3rd), (iii) GSE175648: logFC (8th) and *p*-value (5th).

**Figure 3:**
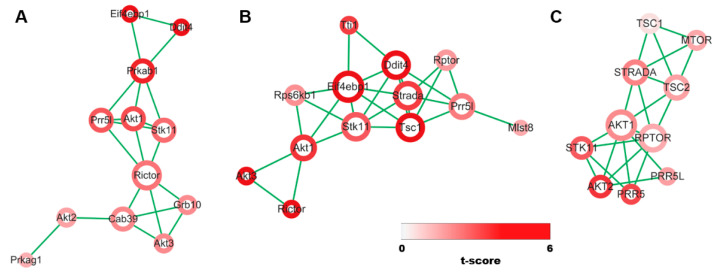
Correlation analysis of mTOR-associated genes across three distinct datasets. (**A**–**C**) Network modules of mTOR-associated genes from three different datasets ((**A**): GSE131735, (**B**): GSE199795, (**C**): GSE175648) whose correlation is computed by Pearson correlation. Circle size is proportional to the number of edges between genes and the color bar represents t-test statistics between two different conditions of the datasets.

To further clarify the interconnection between mTOR-associated genes over the three distinct comparisons, we performed correlation analysis on the genes through Pearson correlation whose cut-off coefficients were GSE131735: 0.997, GSE199795: 0.966, and GSE175648: 0.86 along with visualization. Considering that the number of edges is proportionate to relationship between the genes, mTOR-associated genes whose number of edges are higher than five are (i) AKT1, CAB39, PRKAB1, PRR5L, RICTOR, and STK11 for GSE131735 (Figure 3A); (ii) AKT1, DDIT4, EIF4EBP1, PRR5L, STK11, STRADA, and TSC1 for GSE199795 (Figure 3B); (iii) AKT1, RPTOR, STRADA, and TSC2 for GSE175648 (Figure 3C).

### 3.2. Specimen Experiment: AKT1 Expression in Clear Cell Renal Cell Carcinoma

**Figure 4:**
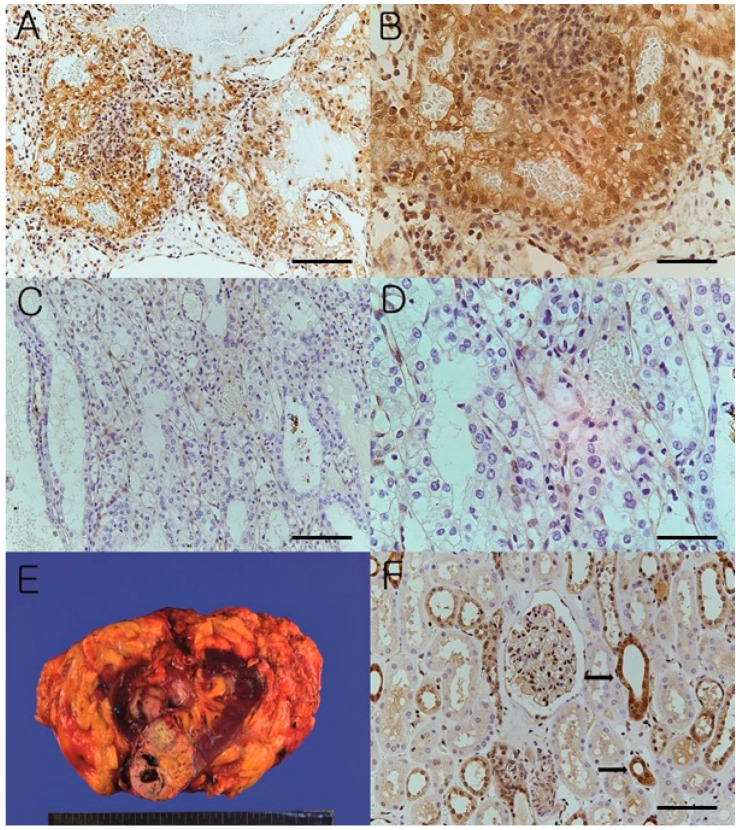
Microscopic and gross features of clear cell renal cell carcinoma. Representative photographs of high AKT1 expression ((**A**), ×200). The high AKT1 expression was seen in both nuclei and cytoplasm of carcinoma cells ((**B**), ×400). Representative photographs of low AKT1 expression. This case showed weakly focal AKT1 expression in only a few carcinoma cells ((**C**), ×200). Magnified view ((**D**), ×400). Representative gross photograph of clear cell renal cell carcinoma. Relatively well circumscribed mass with cystic, solid, and hemorrhagic areas is observed in interpolar area (**E**). Photographs of AKT1 expression in normal kidney ((**F**), ×200). The distal convoluted tubules (Black arrow) and some glomerular mesangial cells showed high AKT1 expression in normal kidney. Scale bar is 20 µm for ×200, and 40 µm for ×400.

**Figure 5:**
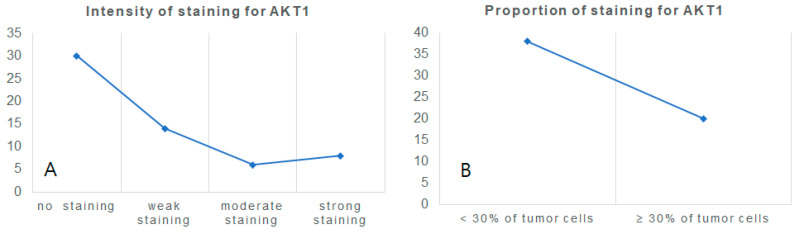
Graphs according to intensity (**A**) and proportion (**B**) regarding AKT1 expression results obtained by immunohistochemistry.

High expression of AKT1 whose expression was examined in both nuclear and cytoplasm was observed in 48.3% (28/58) of the clear cell RCCs while low expression of AKT1 was seen in 51.7% (30/58). The normal kidney showed high expression of AKT1 in distal convoluted tubules (Figure 4). Graphs according to 58 clear cell renal cell carcinoma tissues regarding AKT1 expression intensity and proportion results obtained by immunohistochemistry are shown (Figure 5).

The ages (mean ± standard deviation) of low expression of AKT1 and high expression of AKT1 patients were 60.40 ± 14.122 and 61.00 ± 11.165, respectively, and there was no statistical difference (*p* = 0.859) (Table 1). The gender was male: female 20:10 and 18:10, respectively, without statistical difference (*p* = 0.534).

**Figure 6:**
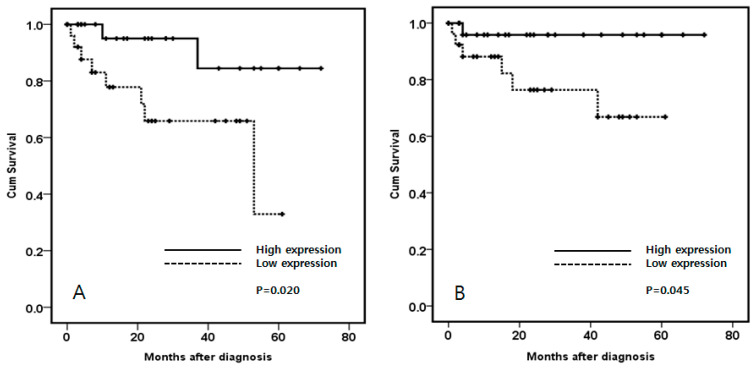
Kaplan–Meier estimates of clear cell kidney cancer patients. Both recurrence-free survival (**A**) and progression-free survival (**B**) show statistically significant differences.

After analyzing the clinicopathological characteristics, WHO/ISUP grade was lower in high expression of AKT1 (*p* = 0.040) while the TNM stage, however, did not show significant results. Recurrence-free survival and progression-free survival were significantly better in high expression of AKT1 than that in low expression of AKT1 (*p* = 0.020, *p* = 0.045, Figure 6).

**Figure 7:**
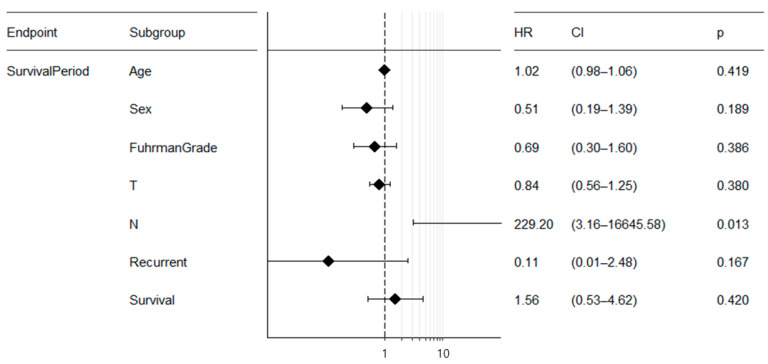
Multivariate analysis by Cox proportional hazard model. Multivariate survival analysis using the technique of Cox regression is applied for multiple covariates (i.e., age, sex, WHO/ISUP grade, T stage, N stage, M stage, recurrent, survival, and progress), possibly interacting each other, and visualized through Forest plot.

Multivariate survival analysis through the Cox proportional hazard model was performed, and the results are presented. In Cox regression analysis, only N stage was observed to be statistically significant among several factors (*p* = 0.013, Figure 7).

## 4. Discussion

PI3K/Akt/mTOR pathway and signaling cascade play an important role in cell growth and metabolism. In the mechanism of cancer, PI3K is closely related to the polyoma middle-T antigen necessary for tumorigenesis in animals [13]. Mutations in PIK3CA, part of the gene for PI3K, have been identified in up to 36% of hepatocellular carcinoma, 26% of breast cancer and 26% of colon cancer [14,15]. AKT1, commonly known as fuselage 1 on v-akt murine thymoma virus tumor gene, is an oncogene and is a member of the PI3K/Akt/mTOR pathway.

AKT regulates essential cellular functions such as apoptosis, migration, proliferation, differentiation and metabolism. AKT affects the expression and/or activity of various angiogenic and anti-angiogenic factors. AKT isotypes, such as AKT1, AKT2 and AKT3, have been suggested as therapeutic targets for angiogenesis-related abnormalities such as tumor or ischemic injury [16].

Hollander et al. reported that AKT1 deficiency suppressed tobacco-induced lung cancer in a study of mice. K-ras mutation, which is closely associated with lung cancer, requires PI3K and mTOR activation. AKT1 deficiency suppressed the mechanism of AKT signaling, inhibiting tumor progression and onset. Human lung cancer cells showing mutated K-ras and reduced AKT1 did not grow in vivo. This is because AKT1-deficient fibroblasts are resistant to epidermal growth factor and transformation by mutant K-ras [17]. Staal SP examined tumors in 224 humans for changes related to AKT1. It was confirmed that this gene was amplified 20-fold in one of the five gastric adenocarcinomas [18].

Recent studies have reported that AKT1 plays a dual role in cell proliferation, migration and invasion depending on the type of cancer cell. In particular, the role of AKT1 in hepatocellular carcinoma and colorectal carcinoma cells is very difficult to understand. Chen et al. reported overexpression of AKT1 significantly enhanced the proliferation rates and promoted the colony formation in both HepG2 and HCT 116 cells. Overexpression of AKT1 induces the movement and infiltration of HepG2 cells, but inhibits the movement and invasion of HCT 116 cells [19]. Subsequent mechanistic investigations revealed that upregulation of AKT1 markedly induced the expression of Bcl-2 and NF-κB. Authors suggest a dual role for AKT1 in tumor cell migration and invasion and highlight the cell type-specific action of Akt1 kinase in regulating cell motility. Similar studies have been shown in studies of breast cancer. When AKT1 was weakly active, it inhibited the proliferation of cancer cells in the laboratory. This is to prevent AKT1 from forming HER2-driven mammary tumor by suppressing negative feedback of RTK signaling [20].

There are also reports that it plays a different role depending on the subtype of AKT. In a previous experimental study with mice, AKT1 promoted de novo tumor formation, while AKT3 inhibited vascular tumor growth. AKT3 inhibits the growth and migration of tumor endothelial cells by inhibiting the activation of translation regulatory kinase S6-Kinase (S6K) by regulating the expression of the Rictor [21].

Some studies demonstrated the potential role of AKT pathway influencing tumorigenesis, tumor progression or prognosis in clear cell RCC and its possibility of therapeutic target [22,23,24]. However, the correlation between AKT1 expression and kidney cancer grade and stage is still controversial. We analyzed the high/low expression of AKT1 and clinicopathological characteristics of kidney cancer. The pathological results, recurrence-free survival and progression-free survival of 28 high expressed patients and 30 low expressed patients were compared. The TNM stage did not show different results according to the degree of AKT1 expression. However, WHO/ISUP grade was different significantly. The higher AKT1 expression showed significant correlation with the lower WHO/ISUP grade. The present study ultimately demonstrated that the patients with high AKT1 expression had a better recurrence-free and progression-free survival rate. These results are similar to those of the aforementioned studies of HCT 116 cells for colorectal cancer and breast cancer.

There are a few limitations in our study. We could not extend high quality research to reaffirm consistent results on other factors in the AKT pathway, expected to be connected to AKT1 closely. Few similarly themed studies on AKT1 expression in clear cell RCC have been reported, and there is still a dearth of research to support the potential role of AKT1. Fan et al. reported that AKT1 was a favorable factor in stage I, while it was a poor factor in stage II and III patients with clear cell RCC [25]. The discrepancy between the findings of some other studies and our findings can be attributed to the refined methodology to determine the degree of AKT1 expression. Furthermore, our result might have been underpowered due to relatively small sample size causing selection bias. To overcome this limitations, large scale, prospective and multicenter trials can verify the significance of AKT1 alteration which may trigger change in prognosis of clear cell RCC.

## 5. Conclusions

The higher AKT1 expression showed the relationship with lower WHO/ISUP grade. It also showed meaningful results for recurrence-free survival and progression-free survival. Patients with high AKT1 expression had better recurrence-free and progression-free survival rate. Therefore, further prospective research in clear cell renal cell carcinoma is needed.

## Figures and Tables

**Table 1:** Baseline characteristic of clear cell renal cell carcinoma patients.

	Low Expression of AKT1 (*n* = 30)	High Expression of AKT1 (*n* = 28)	*p*-Value
Age (mean ± SD)	60.40 ± 14.122	61.00 ± 11.165	0.859
Sex (Male: Female)	20:10	18:10	0.534
WHO/ISUP grade			0.040
Grade I	0	1	
Grade II	8	16	
Grade III	19	9	
Grade IV	3	2	
T stage			0.315
T1	19	23	
T2	4	1	
T3	7	4	
T4	0		
N stage			0.526
N0	28	27	
N1	2	1	
M stage			
M0	28	27	0.526
M1	2	1	

## Data Availability

The original contributions presented in the study are included in the article, further inquiries can be directed to the corresponding author.
